# Supplementary material for: Identity fusion can foster intergroup trust and willingness to cooperate
Source: Commun Psychol. 2025 Aug 15;3:124. doi: 10.1038/s44271-025-00303-9 (PMC12356696; doi:10.1038/s44271-025-00303-9)
Supplement: Supplementary file 2 — Supplementary Information [file 44271_2025_303_MOESM2_ESM.pdf]

Study 1 Supplementary Information

Table S1

Intergroup dyad sample sizes

Social Identity	*n*	% of social identity category
Religious Identity		
Christian	269	34.27
Muslim	516	65.73
Other	31	3.80
Regional/Ethnic Group		
Moro	387	47.43
Lumad	207	25.37
Christian Settler	221	27.08
Other	1	0.12
Political Identity		
UBJP	471	57.7
BAPA	64	7.84
Other	281	65.56

Table S2

Mean (SD) for variables by fusion status to religion, regional group, and political party.

	All Participants	Religion		Regional Group		Political Party	
		Fused	Not Fused	Fused	Not Fused	Fused	Not Fused
Intergroup Cooperation	6.56(0.71)	6.59(0.71)	6.47(0.71)	6.63(0.67)	6.33(0.79)	6.71(0.59)	6.46(0.76)
**Trust of outgroup**							
Religion (Muslims vs. Christians)	5.45(1.20)	5.51(1.22)	5.28(1.12)	5.53(1.21)	5.21(1.16)	5.82(1.14)	5.21(1.18)
Regional Group (Moro vs. Lumad)	5.36(1.38)	5.33(1.49)	5.45(0.97)	5.42(1.41)	5.03(1.16)	5.73(1.36)	5.10(1.34)
Regional Group (Moro vs. CS)	5.29(1.28)	5.32(1.34)	5.15(1.01)	5.38(1.35)	5.03(1.04)	5.72(1.25)	5.00(1.23)
Regional Group (Lumad vs. CS)	5.57(1.20)	5.70(1.20)	5.34(1.17)	5.63(1.29)	5.41(0.90)	5.95(1.16)	5.32(1.16)
Political Party (UBJP vs. BAPA)	5.11(1.38)	5.16(1.43)	4.92(1.19)	5.15(1.42)	4.93(1.20)	5.71(1.29)	4.67(1.28)
**Outgroup Perceptions (i.e., cold-warm)**							
Religion (Muslims vs. Christians)	68.32 (23.83)	72.07 (21.36)	57.11 (27.14)	71.82 (21.65)	57.04 (26.91)	75.61 (21.33)	63.46 (24.19)
Regional Group (Moro vs. Lumad)	71.10 (22.16)	73.30 (20.19)	64.24 (26.35)	73.37 (20.40)	59.32 (26.86)	75.80 (20.48)	67.83 (22.73)
Regional Group (Moro vs. CS)	67.79 (23.17)	71.42 (21.36)	53.63 (24.56)	71.05 (21.48)	58.66 (25.26)	72.63 (22.25)	64.56 (23.23)
Regional Group (Lumad vs. CS)	64.65 (26.14)	68.88 (23.77)	56.97 (28.47)	67.90 (24.02)	55.91 (29.50)	71.14 (23.70)	60.33 (26.83)
Political Party (UBJP vs. BAPA)	66.71 (22.66)	70.07 (21.18)	54.43 (23.71)	68.75 (21.47)	57.60 (25.53)	74.16 (21.37)	61.26 (22.04)
**Outgroup threat perception**							
Religion (Muslims vs. Christians)	1.35(0.79)	1.35(0.81)	1.35(0.70)	1.31(0.76)	1.49(0.84)	1.32(0.80)	1.37(0.78)
Regional Group (Moro vs. Lumad)	1.29(0.76)	1.32(0.80)	1.20(0.58)	1.28(0.77)	1.36(0.67)	1.24(0.74)	1.33(0.77)
Regional Group (Moro vs. CS)	1.39(0.80)	1.35(0.78)	1.52(0.87)	1.30(0.73)	1.62(0.93)	1.30(0.73)	1.45(0.84)
Regional Group (Lumad vs. CS)	1.21(0.60)	1.15(0.51)	1.32(0.73)	1.10(0.39)	1.51(0.90)	1.15(0.51)	1.25(0.65)
Political Party (UBJP vs. BAPA)	1.19(0.54)	1.19(0.53)	1.19(0.58)	1.14(0.45)	1.45(0.81)	1.15(0.55)	1.23(0.54)
**Desire for social distance (relationships) from outgroup**							
Regional Group (Moro vs. Lumad)	2.32(1.22)	2.39(1.29)	2.13(0.96)	2.28(1.26)	2.57(0.98)	2.02(1.22)	2.54(1.18)
Regional Group (Moro vs. CS)	2.33(1.12)	2.37(1.19)	2.16(0.78)	2.32(1.18)	2.33(0.88)	1.96(1.09)	2.58(1.07)
Regional Group (Lumad vs. CS)	1.83(0.97)	1.65(0.94)	2.11(0.96)	1.81(1.05)	1.90(0.50)	1.51(0.86)	2.06(0.99)

**Desire for social distance (land sharing) from outgroup**							
Regional Group (Moro vs. Lumad)	3.53(2.06)	3.31(2.05)	4.22(1.94)	3.47(2.07)	3.82(1.98)	3.22(2.04)	3.75(2.05)
Regional Group (Moro vs. CS)	2.97(1.79)	2.92(1.81)	3.19(1.71)	2.99(1.84)	2.93(1.61)	2.57(1.76)	3.26(1.77)
Regional Group (Lumad vs. CS)	3.75(2.13)	3.53(2.18)	4.11(2.01)	3.85(2.12)	3.34(2.17)	3.23(1.97)	4.14(2.17)

Table S3

The relationship between identity fusion and outgroup trust moderated by threat, controlling for group membership

Terms	*df*	*f*	*p*	*Adj. R^2*	*b*	*CIs*	*t*	*p*
Model 1: Muslim & Christian Filipinos	**780**	**24.35**	**<.001**	**0.11**				
**Intercept**					**5.59**	**[5.4, 5.78]**	**58.59**	**<.001**
**Fusion to religion**					**0.29**	**[0.11, 0.48]**	**3.1**	**.002**
**Religious group [Reference = Christians]**					**-0.54**	**[-0.71, -0.37]**	**-6.16**	**<.001**
**Outgroup threat**					**-0.33**	**[-0.51, -0.15]**	**-3.53**	**<.001**
Fusion*Threat					0.04	[-0.17, 0.24]	0.38	.703
Model 2: Lumad & CS	**415**	**2.53**	**.028**	**0.01**				
**Intercept**					**5.53**	**[5.29, 5.77]**	**45.48**	**<.001**
Fusion to regional group					0.16	[-0.12, 0.44]	1.14	.253
Regional group [Reference = CS]					-0.2	[-0.44, 0.05]	-1.58	.115
**Outgroup threat**					**-0.16**	**[-0.31, -0.01]**	**-2.1**	**.036**
Fusion*Threat					0.01	[-0.24, 0.27]	0.1	.919
Model 3: Moro & CS	**603**	**19.19**	**<.001**	**0.11**				
**Intercept**					**5.44**	**[5.23, 5.66]**	**49.34**	**<.001**
**Fusion to regional group**					**0.46**	**[0.23, 0.69]**	**3.9**	**<.001**
**Regional group [Reference = CS]**					**-0.76**	**[-0.97, -0.55]**	**-7.16**	**<.001**
**Outgroup threat**					**-0.3**	**[-0.46, -0.13]**	**-3.55**	**<.001**
Fusion*Threat					0.16	[-0.05, 0.37]	1.53	.126
Model 4: Moro & Lumad	**585**	**25.98**	**<.001**	**0.15**				
**Intercept**					**5.66**	**[5.36, 5.96]**	**36.7**	**<.001**
Fusion to regional group					0.28	[0, 0.56]	1.95	.052
**Regional group [Reference = Lumad]**					**-0.82**	**[-1.04, -0.6]**	**-7.36**	**<.001**
**Outgroup threat**					**-0.6**	**[-0.89, -0.3]**	**-3.98**	**<.001**
**Fusion*Threat**					**0.39**	**[0.07, 0.7]**	**2.42**	**.016**

Model 5: UBJP & BAPA	**530**	**26.83**	**<.001**	**0.16**				
**Intercept**					**5.02**	**[4.69, 5.34]**	**30.42**	**<.001**
**Fusion to regional group**					**1.02**	**[0.8, 1.24]**	**9.19**	**<.001**
**Political group [Reference = BAPA]**					**-0.38**	**[-0.71, -0.04]**	**-2.23**	**.026**
**Outgroup threat**					**-0.26**	**[-0.41, -0.12]**	**-3.64**	**<.001**
**Fusion*Threat**					**0.23**	**[0.01, 0.44]**	**2.03**	**.043**

Notes: CS is the abbreviation of "Christian Settler". The reference group for the fusion term in all models was non-fused group members. Significant values at the .05 level are bolded.

Supplementary Note 1:

Identity fusion and outgroup perceptions on social distance

We examined whether identity fusion positively predicted a desire for social distance (both relational and land sharing) when threat was high, or outgroup perceptions were positive. Given that much of the intergroup conflict in the BARMM is related to land disputes, examining the role of threat in influencing land sharing was especially intriguing. Interaction analyses suggested that the explicit measure of threat typically moderated the relationship between fusion and *land sharing* social distance (see Table 4), whereas outgroup perceptions moderated the relationship between fusion and *relational* social distance (see Table 5). Fused participants generally desired more relational social distance (e.g., an unwillingness to accept outgroup members as close relations) than unfused participants when outgroups were perceived less positively, and more land sharing social distance (i.e., an unwillingness to sell or rent land to the outgroup) than unfused participants when outgroups were perceived as more of a threat. The only exception to this trend was the lack of a significant threat moderation of fusion on land sharing in the Lumad-Christian Settler dyad, which may be explained by the unusually low number of Lumads that perceived Christian Settlers as a threat (94% Lumads reported the minimum score). This model was also slightly underpowered for a small interaction effect size[1] (n = 317).

---

[1] This was due to a programming error in which only slightly more than half of all Christian Settler participants were shown the social distance items, affecting the Lumad-Christian Settler Social Distance models.

Table S4

The relationship between identity fusion and social distance [relational] moderated by outgroup threat and outgroup perceptions, controlling for group membership

Terms	*df*	*f*	*p*	*Adj. R^2*	*b*	*Cis*	*t*	*p*
Threat Models								
Model 1: Lumad & CS	**312**	**3.35**	**.006**	**0.03**				
**Intercept**					**1.72**	**[1.45, 1.98]**	**12.92**	**<.001**
Fusion to regional group					-0.19	[-0.47, 0.09]	-1.36	.173
**Regional group [Reference = CS]**					**0.42**	**[0.19, 0.65]**	**3.61**	**<.001**
Outgroup threat					0.03	[-0.23, 0.29]	0.21	.837
Fusion* Threat					-0.01	[-0.34, 0.32]	-0.05	.964
Model 2: Moro & CS	**495**	**33.96**	**<.001**	**0.21**				
**Intercept**					**2.27**	**[2.08, 2.46]**	**23.07**	**<.001**
Fusion to regional group					-0.09	[-0.31, 0.13]	-0.79	.431
**Regional group [Reference = CS]**					**0.5**	**[0.4, 0.6]**	**9.61**	**<.001**
**Outgroup threat**					**0.28**	**[0.08, 0.48]**	**2.77**	**.006**
Fusion*Threat					0.02	[-0.21, 0.24]	0.15	.883
Model 3: Moro & Lumad	**585**	**32.46**	**<.001**	**0.18**				
Intercept					1.91	[1.65, 2.17]	14.24	<.001
Fusion to regional group					-0.19	[-0.44, 0.05]	-1.54	.123
**Regional group [Reference = Lumad]**					**0.89**	**[0.7, 1.09]**	**9.22**	**<.001**
**Outgroup threat**					**0.26**	**[0.01, 0.52]**	**2.03**	**.043**
Fusion*Threat					-0.04	[-0.31, 0.23]	-0.29	.771
Outgroup Perceptions Models								
Model 1: Lumad & CS	**315**	**22.75**	**<.001**	**0.21**				
**Intercept**					**1.66**	**[1.38, 1.93]**	**11.92**	**<.001**
Fusion to regional group					0.02	[-0.27, 0.31]	0.14	.885
**Regional group [Reference = CS]**					**0.31**	**[0.11, 0.52]**	**2.98**	**.003**
Outgroup perceptions					-0.16	[-0.37, 0.05]	-1.47	.143

**Fusion*Perceptions**					**-0.35**	**[-0.6, -0.11]**	**-2.84**	**.005**
Model 2: Moro & CS	**495**	**33.9**	**<.001**	**0.21**				
**Intercept**					**2.25**	**[2.04, 2.46]**	**20.88**	**<.001**
Fusion to regional group					-0.03	[-0.27, 0.2]	-0.28	.781
**Regional group [Reference = CS]**					**0.51**	**[0.41, 0.61]**	**9.67**	**<.001**
Outgroup perceptions					-0.11	[-0.29, 0.08]	-1.15	.25
**Fusion*Perceptions**					**-0.24**	**[-0.46, -0.03]**	**-2.19**	**.029**
Model 3: Moro & Lumad	**589**	**57.38**	**<.001**	**0.28**				
**Intercept**					**1.88**	**[1.61, 2.14]**	**13.89**	**<.001**
Fusion to regional group					-0.09	[-0.34, 0.15]	-0.74	.46
**Regional group [Reference = Lumad]**					**0.86**	**[0.68, 1.04]**	**9.49**	**<.001**
Outgroup perceptions					-0.11	[-0.29, 0.06]	-1.30	.195
**Fusion* Perceptions**					**-0.42**	**[-0.62, -0.22]**	**-4.11**	**<.001**

Notes: CS is the abbreviation of "Christian Settler"; SD is the abbreviation of "Social Distance". The reference group for the fusion term in all models was non-fused group members. Significant values at the .05 level are bolded.

Table S5

The relationship between identity fusion and social distance [land sharing] moderated by outgroup threat and outgroup perceptions, controlling for group membership

Terms	*df*	*f*	*p*	*Adj. R^2*	*b*	*Cis*	*t*	*p*
Threat Models								
Model 1: Lumad & CS	**312**	**14.34**	**<.001**	**0.14**				
**Intercept**					**2.65**	**[2.11, 3.18]**	**9.68**	**<.001**
Fusion to regional group					-0.03	[-0.6, 0.55]	-0.09	.929
**Regional group [Reference = CS]**					**1.64**	**[1.17, 2.11]**	**6.81**	**<.001**
Outgroup threat					-0.34	[-0.88, 0.2]	-1.24	.214
Fusion* Threat					-0.06	[-0.74, 0.63]	-0.16	.871
Model 2: Moro & CS	**495**	**4.56**	**<.001**	**0.03**				
**Intercept**					**2.68**	**[2.26, 3.11]**	**12.41**	**<.001**
Fusion to regional group					-0.03	[-0.41, 0.36]	-0.13	.893
**Regional group [Reference = CS]**					**0.44**	**[0.06, 0.82]**	**2.3**	**.022**
**Outgroup threat**					**-0.39**	**[-0.74, -0.03]**	**-2.16**	**.031**
**Fusion*Threat**					**0.64**	**[0.24, 1.04]**	**3.15**	**.002**
Model 3: Moro & Lumad	**585**	**15.28**	**<.001**	**0.09**				
**Intercept**					**4.7**	**[4.23, 5.17]**	**19.81**	**<.001**
**Fusion to regional group**					**-0.47**	**[-0.91, -0.04]**	**-2.14**	**.033**
**Regional group [Reference = Lumad]**					**-1.19**	**[-1.53, -0.86]**	**-6.95**	**<.001**
Outgroup threat					-0.44	[-0.89, 0.02]	-1.9	.058
**Fusion*Threat**					**0.7**	**[0.21, 1.18]**	**2.84**	**.005**
Outgroup Perceptions Models								
Model 1: Lumad & CS	**315**	**29.18**	**<.001**	**0.26**				
**Intercept**					**2.17**	**[1.59, 2.75]**	**7.34**	**<.001**
**Fusion to regional group**					**0.78**	**[0.16, 1.39]**	**2.5**	**.013**
**Regional group [Reference = CS]**					**1.49**	**[1.05, 1.93]**	**6.72**	**<.001**
**Outgroup perceptions**					**-0.78**	**[-1.24, -0.33]**	**-3.4**	**<.001**

Fusion*Perceptions					-0.06	[-0.58, 0.46]	-0.22	.830
Model 2: Moro & CS	**495**	**11.14**	**<.001**	**0.08**				
**Intercept**					**2.62**	**[2.17, 3.08]**	**11.34**	**<.001**
Fusion to regional group					0.13	[-0.28, 0.53]	0.62	.535
**Regional group [Reference = CS]**					**0.44**	**[0.07, 0.81]**	**2.34**	**.020**
Outgroup perceptions					-0.03	[-0.35, 0.29]	-0.16	.872
**Fusion*Perceptions**					**-0.6**	**[-0.97, -0.22]**	**-3.09**	**.002**
Model 3: Moro & Lumad	**589**	**33.18**	**<.001**	**0.18**				
**Intercept**					**4.43**	**[3.95, 4.9]**	**18.26**	**<.001**
Fusion to regional group					-0.09	[-0.53, 0.35]	-0.4	.687
**Regional group [Reference = Lumad]**					**-1.24**	**[-1.56, -0.92]**	**-7.63**	**<.001**
**Outgroup perceptions**					**-0.55**	**[-0.86, -0.24]**	**-3.48**	**<.001**
Fusion*Perceptions					-0.2	[-0.56, 0.16]	-1.09	.275

Study 2 Supplementary Information

Table S6

Mean (SD) for key variables overall and by country.

	All Participants	Gambia	Tanzania	Pakistan	Uganda
Identity Fusion	6.06(1.00)	5.75(1.03)	6.17(0.88)	5.92(1.13)	6.27(0.83)
Willingness to Cooperate	5.20(1.83)	5.14(1.62)	5.38(1.98)	5.42(1.64)	4.89(1.94)
Outgroup Threat	3.28(1.91)	3.52(1.69)	3.44(2.28)	3.67(1.73)	2.65(1.76)
Outgroup Perceptions	4.57(1.19)	4.84(1.19)	4.52(1.40)	4.79(1.03)	4.27(1.13)
Historical Threat	3.94(1.97)	4.92(1.68)	3.61(2.17)	4.36(1.73)	3.28(1.88)
Ingroup Benefit	4.81(2.05)	1.91(1.33)	5.43(1.67)	5.58(1.49)	4.98(1.86)
Zero-Sum Perceptions	3.22(1.93)	2.98(1.67)	3.74(2.28)	3.51(1.76)	2.69(1.80)

Table S7

The relationships between identity fusion, identification, and willingness to cooperate moderated by outgroup threat, outgroup perceptions, historical threat, ingroup benefit, and zero-sum perceptions.

Terms	*Marginal* R^2	*Conditional* R^2	*df*	*B*	*CIs*	*t*	*p*
Model 1: Threat	0.1	0.15					
**Intercept**			**3.07**	**5.22**	**[4.79, 5.66]**	**26.57**	**<.001**
Identity fusion			1503.71	-0.05	[-0.16, 0.06]	-0.86	.389
**Identification**			**1501.49**	**0.28**	**[0.17, 0.39]**	**4.98**	**<.001**
**Threat**			**1503.98**	**-0.48**	**[-0.57, -0.39]**	**-10.66**	**<.001**
Fusion*Threat			1501.96	0.05	[-0.05, 0.16]	0.99	.323
**ID*Threat**			**1501.36**	**-0.22**	**[-0.33, -0.11]**	**-3.89**	**<.001**
Model 2: Perceptions	0.24	0.26					
**Intercept**			**2.85**	**5.21**	**[4.94, 5.47]**	**43.28**	**<.001**
Identity fusion			1489.65	0.01	[-0.09, 0.11]	0.12	.906
**Identification**			**1488.92**	**0.15**	**[0.05, 0.25]**	**2.84**	**.005**
**Perceptions**			**1482.21**	**0.85**	**[0.77, 0.93]**	**20.29**	**<.001**
Fusion* Perceptions			1488.44	0.05	[-0.05, 0.14]	0.98	.328
ID* Perceptions			1487.60	0.09	[-0.01, 0.18]	1.73	.083
Model 3: Hist. Threat	0.05	0.08					
**Intercept**			**3.11**	**5.22**	**[4.86, 5.58]**	**32.04**	**<.001**
Identity fusion			1506.90	-0.05	[-0.16, 0.07]	-0.82	.414
**Identification**			**1504.83**	**0.30**	**[0.18, 0.41]**	**5.15**	**<.001**
**Hist. Threat**			**1469.59**	**-0.20**	**[-0.29, -0.11]**	**-4.23**	**<.001**
Fusion*Hist. Threat			1506.31	< .01	[-0.11, 0.11]	0.02	.984
**ID*Hist. Threat**			**1504.70**	**-0.21**	**[-0.32, -0.09]**	**-3.48**	**<.001**
Model 4: Benefit	0.38	0.61					
**Intercept**			**2.97**	**5.42**	**[4.25, 6.61]**	**10.1**	**.002**
Identity fusion			1508.31	-0.02	[-0.11, 0.07]	-0.4	.687
**Identification**			**1508.24**	**0.13**	**[0.04, 0.22]**	**2.76**	**.006**
**Benefit**			**1507.80**	**1.36**	**[1.27, 1.45]**	**29.42**	**<.001**
**Fusion*Benefit**			**1508.60**	**0.10**	**[0.02, 0.19]**	**2.32**	**.020**
Identification*Benefit			1508.11	0.01	[-0.08, 0.1]	0.28	.783
Model 5: Zero-Sum	0.11	0.16					
**Intercept**			**3.05**	**5.22**	**[4.79, 5.65]**	**26.63**	**<.001**

Identity fusion	1503.49	< 0.01	[-0.11, 0.1]	-0.08	.936
**Identification**	**1501.54**	**0.23**	**[0.12, 0.34]**	**4.15**	**<.001**
**Zero-sum**	**1503.82**	**-0.52**	**[-0.61, -0.43]**	**-11.48**	**<.001**
Fusion*Zero-sum	1501.59	-0.07	[-0.19, 0.04]	-1.28	.200
**ID*Zero-sum**	**1501.96**	**-0.13**	**[-0.24, -0.02]**	**-2.22**	**.027**

Notes: Significant values at the .05 level are bolded.
